# Review of malaria situation in Cameroon: technical viewpoint on challenges and prospects for disease elimination

**DOI:** 10.1186/s13071-019-3753-8

**Published:** 2019-10-26

**Authors:** Christophe Antonio-Nkondjio, Cyrille Ndo, Flobert Njiokou, Jude D. Bigoga, Parfait Awono-Ambene, Josiane Etang, Albert Same Ekobo, Charles S. Wondji

**Affiliations:** 1 Laboratoire de Recherche sur le Paludisme, Organisation de Coordination pour la lutte contre les Endémies en Afrique Centrale (OCEAC), B. P.288 Yaoundé, Cameroun; 2 Department of Biological Sciences, Faculty of Medicine and Pharmaceutical Sciences, University of Douala, P.O. Box 24157, Douala, Cameroon; 3 Centre for Research in Infectious Disease (CRID), P.O. Box 13591, Yaoundé, Cameroon; 4 Faculty of Sciences, University of Yaoundé I, P.O. Box 337, Yaoundé, Cameroon; 5 Vector Biology Liverpool School of Tropical medicine Pembroke Place, Liverpool, UK; 6 Laboratory for Vector Biology and control, National Reference Unit for Vector Control, The Biotechnology Center, Nkolbisson-University of Yaounde I, P.O. Box 3851, Messa, Yaounde, Cameroon; 7 Department of Biochemistry, Faculty of Science, University of Yaounde I, Yaounde, Cameroon; 8 Institute for Insect Biotechnology, Justus Liebig University Gießen, Winchester Str. 2, 35394 Gießen, Germany

**Keywords:** Malaria, *Plasmodium*, Vector control, Drug resistance, Insecticide resistance, *Anopheles*, Cameroon

## Abstract

Malaria still has a devastating impact on public health and welfare in Cameroon. Despite the increasing number of studies conducted on disease prevalence, transmission patterns or treatment, there are to date, not enough studies summarising findings from previous works in order to identify gaps in knowledge and areas of interest where further evidence is needed to drive malaria elimination efforts. The present study seeks to address these gaps by providing a review of studies conducted so far on malaria in Cameroon since the 1940s to date. Over 250 scientific publications were consulted for this purpose. Although there has been increased scale-up of vector control interventions which significantly reduced the morbidity and mortality to malaria across the country from a prevalence of 41% of the population reporting at least one malaria case episode in 2000 to a prevalence of 24% in 2017, the situation is not yet under control. There is a high variability in disease endemicity between epidemiological settings with prevalence of *Plasmodium* parasitaemia varying from 7 to 85% in children aged 6 months to 15 years after long-lasting insecticidal nets (LLINs) scale-up. Four species of *Plasmodium* have been recorded across the country: *Plasmodium falciparum*, *P. malariae*, *P. ovale* and *P. vivax*. Several primate-infecting *Plasmodium* spp. are also circulating in Cameroon. A decline of artemisinin-based combinations therapeutic efficacy from 97% in 2006 to 90% in 2016 have been reported. Several mutations in the *P. falciparum* chloroquine resistance (*Pfcrt)* and *P. falciparum* multidrug resistance 1 (*Pfmdr1*) genes conferring resistance to either 4-amino-quinoleine, mefloquine, halofanthrine and quinine have been documented. Mutations in the *Pfdhfr* and *Pfdhps* genes involved in sulfadoxine-pyrimethamine are also on the rise. No mutation associated with artemisinin resistance has been recorded. Sixteen anopheline species contribute to malaria parasite transmission with six recognized as major vectors: *An. gambiae*, *An. coluzzii*, *An. arabiensis*, *An. funestus*, *An. nili* and *An. moucheti.* Studies conducted so far, indicated rapid expansion of DDT, pyrethroid and carbamate resistance in *An. gambiae*, *An. coluzzii*, *An. arabiensis* and *An. funestus* threatening the performance of LLINs. This review highlights the complex situation of malaria in Cameroon and the need to urgently implement and reinforce integrated control strategies in different epidemiological settings, as part of the substantial efforts to consolidate gains and advance towards malaria elimination in the country.

## Background

Malaria is still an important public health threat in Cameroon with the whole country exposed to the risk of transmission [1, 2]. Although significant progress has been made in the recent past, the disease remains prevalent with a high number of suspected cases in health care facilities varying between 3.3–3.7 million per year [1]. Malaria parasite transmission is highly heterogeneous with high and perennial parasite transmission occurring in the forest, coastal and humid savanna areas and low parasite transmission in highlands and seasonal parasite transmission in sahelian and dry savanna areas [3]. *Plasmodium falciparum* is the main parasite responsible for over 95% of the cases [4]. Other human-infecting *Plasmodium* species circulating in the country include *P. malariae*, *P. ovale* and *P vivax* [5]. The latter parasite species which was thought to be absent from West and Central Africa in more recent evolutionary times, has now been reported in the country [6–8], highlighting the changing pattern of malaria in Cameroon. However, the epidemiological role of this species as well as local vector species competence for this parasite is still to be determined. Up to 52 anopheline species have been reported in the country so far, with 16 recognized as main or secondary vectors [9–11]. Six of the species are among the most efficient vectors in sub-Saharan Africa, namely, *An. gambiae* (*s.s.*), *An. coluzzii*, *An. arabiensis*, *An. funestus*, *An. nili* and *An. moucheti* [11, 12].

Vector control has been a vital component of malaria prevention and control, relying mainly on the use of long-lasting insecticidal nets (LLINs). Since 2000, Cameroon has benefited from the support of various international partners to implement malaria control interventions [9, 13]. Over 20 million LLINs have so far been freely distributed to the population through several campaigns [1], with the support of partners (e.g. the Global Fund). Although the coverage rate of the population is still below the target of the Ministry of Health (> 80% of the households having one net for two persons), it is estimated that between 2000 and 2015, the scale-up of treated bednets across the country resulted in a significant decrease in the prevalence of malaria reported cases from 41% to 24.3%, and 54% decrease in malaria related mortality (from about 13,000 to 6000 per year) [1].

In the northern regions of the country where malaria parasite transmission is seasonal and prone to frequent eruptions of epidemics, seasonal chemoprevention has been introduced and targets mostly children [1]. In 2017, Cameroon was selected as a US President’s Malaria Initiative (PMI) focus country. The PMI programme, which will focus essentially in the North and Far North regions, will support the procurement of over 250,000 LLINs for routine distribution to pregnant women during antenatal care and will undertake indoor residual spraying (IRS) trials to foster malaria elimination in this part of the country [14]. A third nationwide free distribution of over 15 million LLINs to the population is planned for 2019 [1]. Other interventions are being piloted in other epidemiological settings of the country such as larviciding in the city of Yaoundé and the PADY (Projet d’Assainissement de Yaoundé) programme focusing on hygiene and sanitation in Yaoundé [15, 16]. Concerning malaria treatment, several programmes including case management are undertaken regularly to improve the management of malaria cases and tracking of drug resistance [17–23]. All these efforts, if well-coordinated, could further improve malaria control in Cameroon. Thus, there is still a need to further probe into the understanding of malaria epidemiology and transmission ecology for informed decision-making and to better coordinate control intervention strategies across the country.

Although there have been several studies on malaria epidemiology, case management, parasite prevalence, drug resistance, vectors distribution, bionomics, role in malaria parasite transmission or insecticide resistance since the 1950s, little has been done to assess the impact of control interventions on disease transmission. Also, there are still not enough reviews summarising previous data in order to identify gaps in knowledge or to document recent evolution and dynamics of the vectors or the parasites. Such information is essential for the management of control programmes and scale-up of new or supplemental intervention strategies.

The objective of the present review is to collate information from previous studies in order to better appraise the complexity of malaria situation and evidence in order to guide efforts towards malaria elimination in Cameroon. Although strengthening the health care system is an important requirement to achieve malaria elimination this has not been included in the present review which limits itself to an assessment of technical challenges and interventions.

## Data retrieval

Information on malaria in Cameroon were extracted from published reports. Online bibliographic databases including PubMed, Google and Google Scholar were used to search for information. Terms used to guide these searches included “malaria”, “parasite”, “drug resistance”, “vector control”, “*Plasmodium*”, “LLINs”, “insecticide resistance”, “*Anopheles*”, “Cameroon”, “susceptibility”, “case management” “Yaoundé” and “Douala”. The search period included 1940 to 2019. The search resulted in 1029 articles. Over 750 papers were excluded because they were not on malaria or not reporting data from Cameroon.

Information extracted from each selected published study were entered into a Microsoft Excel spreadsheet for easy access and data analysis. Information registered included authors names, the year of the study, methods and main findings.

## Situation of malaria in Cameroon

**Fig. 1:**
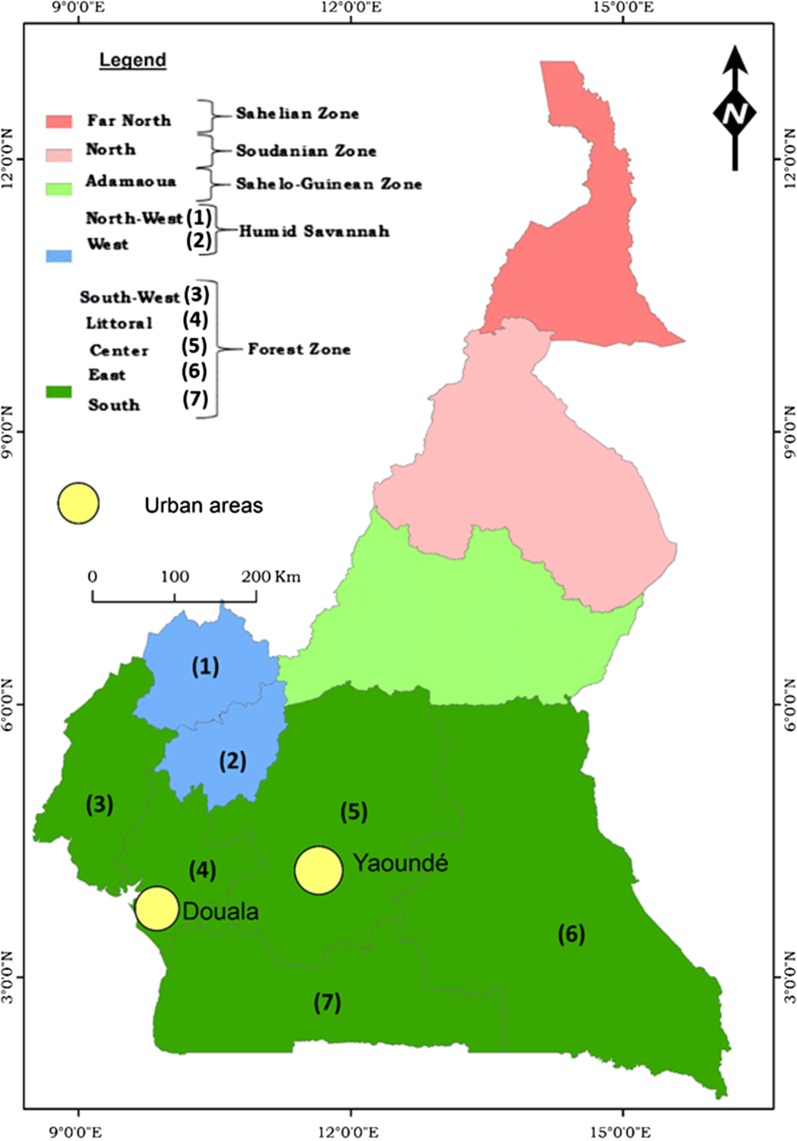
A map of Cameroon showing climatic and administrative divisions

Cameroon is situated in central Africa, within the Gulf of Guinea at a latitude between 2–13°N and a longitude between 9–16°E. It has a surface area of approximately 475,000 km^2^ with a population of about 24 million [24]. It is bordered to the West by Nigeria, to the North and East by Chad, to the East by Central African Republic and to the South by Congo, Gabon and Equatorial Guinea [25]. The country also has a coastal border of about 400 km with the Atlantic Ocean. Administratively, Cameroon is divided into 10 administrative regions covering different ecological domains (Fig. 1). Data from the demographic and health survey (DHS) and from the malaria indicator survey (MIS), indicated vegetation and altitude as important predictors of the geographical distribution of malaria in Cameroon [2]. During the last decade an increase in temperature of 0.4 °C and decrease in rainfall of 10–20% have been reported, compared to the period 1951–1980 [26]. Across sub-Saharan Africa, similar projections have been reported with an increase in temperature of 1.5 °C above the 1951–1980 baseline level [27]. Although this situation coincided with a certain number of events such as frequent reports of dengue cases in the country [28, 29], outbreaks of chikungunya and yellow fever in Cameroon and neighbouring countries [30–32] or invasion of Cameroon by *Aedes albopictus* mosquitoes originating from Asia [33, 34], there have not been many studies assessing the direct relationship between vector-borne diseases dynamics and changing climate conditions in Cameroon. This deserves further investigation in the light of some recent reviews [35–39].

The Far North region belongs to the sahelian domain characterized by hot and dry weather with annual rainfall never exceeding 700 mm/year. According to malaria stratification the Far North region belongs to a hyperendemic malaria stratum with seasonal malaria parasite transmission prone to cyclic outbreaks. The Far North region is one of the most densely populated regions in the country, with a population estimated at 3.9 million inhabitants occupying a surface area of 34,263 km^2^ (Table 1) [40]. The fast demographic growth of the area, deforestation and desertification, deeply affected the landscape of the area which is now witnessing extended dry seasons with a reduction in crops yields and livestock productivity [41].

**Table 1:** Population repartition and bed net coverage in the different regions of Cameroon

Regions	Population in 1987	Population in 2015	Size (km^2^)	Inhabitants/km^2^	Bednet coverage in 2013^a^	Bednet coverage in 2017^a^	Bednet usage in 2017^b^
Far North	1,855,695	3,945,168	34,263	90.8	83.4	80.9	35.4
North	832,165	2,410,936	66,090	25.5	75.3	93.9	83.1
Adamaoua	495,185	1,183,551	63,701	13.9	70.9	81.8	80.1
North-West	1,237,348	1,950,667	17,300	99.9	79.2	66.9	56.0
West	1,339,791	1,906,831	13,892	123.8	45.0	74.0	57.3
Centre	1,651,600	4,098,592	68,953	44.9	59.8	78.5	61.2
Littoral	1,352,833	3,309,558	20,248	124.0	53.5	73.0	59.3
South	373,798	745,198	47,191	13.4	68.7	83.7	54.3
South-West	838,042	1,534,232	25,410	51.8	67.1	69.7	59.1
East	517,198	832,869	109,002	7.1	58.6	77.2	55.6
Cameroon	10,493,655	21,917,602	475,000	46.14	65.6	76.6	58.3

^a^Proportion of households with at least one LLIN

^b^The proportion of people who slept in a LLIN the previous night

In this region, frequent malaria epidemics occur during the rainy season which last 2–3 months [42]. Surveys conducted before the implementation of LLINs in the sites of Koza, Yagoua and Maga, indicated the prevalence of *P. falciparum* parasitaemia in children (*n* = 924) aged between 2 and 9 years-old, varying from 8.5% at the end of the dry season to 40.8% during the rainy season [43, 44]. A similar pattern was recorded in other sites of the region in Mahouda, Simatou, Guividig and Farahoulou with malaria prevalence varying from 10% (62/616) to 17.2% (109/632) [44]. Following LLINs scale-up, a decrease in the prevalence in children to 7.3–9.2% (*n* = 341) was recorded in 2017 [45]. However, the region still reported the highest number of malaria cases in 2015 in the country according to the NMCP annual report [3]. Entomological inoculation rate (EIR) was not reported to vary much before and after LLINs scale-up it is estimated to range between 2.4–24.0 infective bites/person/month during the rainy season, with *An. arabiensis* as the main vector species [46, 47]. The difference between reported entomological and epidemiological data may be due to the fact that the studies cited in the present review were not conducted in the same sites.

The North region is located within the dry savanna domain and is characterized by a rainy season lasting 3–5 months with annual rainfall reaching 1000 mm/year. According to malaria stratification, this area belongs to a hyperendemic malaria stratum with seasonal malaria parasite transmission prone to cyclic outbreaks (transmission period could be extended compare to the Far North region). The population in the area is estimated at 2.4 million inhabitants on a surface area of 66,090 km^2^. The region population more than doubled during the last decades due in part to migration of population affected by droughts or displaced by the Boko Haram insurgency. Before LLINs scale-up in the country, malaria parasite prevalence in children of 1–15 years-old, was estimated to vary between 6.5–30.7% (*n* = 655) during cross-sectional surveys in the health districts of Lagdo [46, 48]. The EIR was found to vary between 2.7–36.5 infected bites/person/month [49, 50]. After LLINs scale-up, average malaria parasite prevalence levels of 30.4% [varying significantly from 28.6% (798/2795) for net users and 35% (243/694) for non-net users] was recorded in the health districts of Garoua, Pitoa and Mayo-Oulo in children of 6 months to 5 years-old (Table 2) [51]. Intense transmission was found to occur during the rainy season with estimates varying from 24.5 to 60 infective bites/person/month in the health districts of Lagdo, Garoua, Pitoa, Mayo Mbocki and Mayo Oulo [49, 50, 52, 53]. The increase in the EIR rate recorded for this region could derive from the fact that entomological surveys were undertaken in different sites before and after LLINs scale-up. It is possible that localities scoring high EIR had much higher rate before LLINs scale-up. Main vectors species in the area are *An. arabiensis*, *An. gambiae* and *An. funestus*. Other species playing a role in malaria parasite transmission are *An. pharoensis*, *An. coluzzii*, *An. rufipes* and *An. ziemanni* [50, 52].

**Table 2:** Summary of entomological and epidemiological data according to regions before and after LLINs scale-up

Region	Before LLINs scale-up		After LLINs scale-up		Transmission pattern	Main vector	References
	EIR	Epidemiological data (%)	EIR	Epidemiological data^*b*^ (%)			
Far North	6.0–20.0 ib/p/m	8.5–40.8^b^	6.0–20.0 ib/p/m	7.3–9.2^b^	Seasonal	*An. arabiensis*	[42–47]
North	2.7–36.5 ib/p/m	6.5–30.7^a^	24.5–60.0 ib/p/m	28.6–35^c^	Seasonal	*An. arabiensis*; *An. gambiae* (*s.s.*); *An. funestus*	[46, 49–53]
Adamaoua	100 ib/p/yr	17.5^b^		8.1–10.6^b^	Perennial with high intensity	*An. gambiae* (*s.s.*); *An. coluzzii*; *An. funestus*	[55–59]
North-West and West	62.8–90.5 ib/p/yr	25.0–53.2^a^	2.2–11.0 ib/p/yr	9.3–22.4^a^	Perennial with low intensity	*An. Gambiae* (*s.s.*); *An. funestus*	[8, 58, 60–64]
Littoral, South-West, Centre, South and East	149.0–287.0 ib/p/yr	35.0–85.4^a^	0.7–1.4 ib/p/m	9.0–56.2^a^	Perennial with high intensity	*An. gambiae* (*s.s.*); *An. coluzzii*; *An. funestus*; *An. moucheti*; *An. nili*	[18, 44, 49, 58, 64–95]
Yaoundé and Douala	3.0–33.0 ib/p/yr	35.0^a^	0–90.0 ib/p/yr	35.0^b^	Seasonal	*An. gambiae* (*s.s.*); *An. coluzzii*	[66, 96–103]

^a^Prevalence of malaria parasite in asymptomatic children of 1 to 15 years

^b^Prevalence of malaria parasite in asymptomatic children of 2 to 9 years

^c^Prevalence of malaria parasite in asymptomatic children of 6 months to 5 years-old

*Abbreviations*: EIR, entomological inoculation rate; ib/p/m, infected bites per person per month; ib/p/yr, infected bites per person per year

The Adamaoua region situated mid-way between the North and the Centre regions, is dominated by a landscape above 1000 m and is characterized by humid savannah with one rainy season lasting over 6 months with a moderate climate and precipitation which amounts to 1500 mm/year. In some parts of the region, daily average temperatures could be as low as 20 °C part of the year. The region could be classified as belonging to a mesoendemic stratum with perennial malaria parasite transmission due to the abundance of rivers and lakes. The population of the area is about 1.18 million inhabitants living on a surface area of 63,701 km^2^ [40]. The region as well as the North, Far-North and East regions have several displaced camps for refugees or displaced persons coming from neighbouring countries, such as, Nigeria, Chad or the Central African Republic [54]. Yet there is still not enough information on how this influx of people has influenced local disease epidemiology which still deserves further investigation. Before LLINs, scale-up malaria parasite prevalence in 2–9 year-old children was 17.5% (*n* = 724) [55] and entomological inoculation rate (EIR) was 100 infected bites/person/year [56, 57]. After LLINs scale-up in 2017, parasites rates in febrile children of 2–9 years-old were reported to vary from 8.1 to 10.6% (*n* = 315) [58]. High *Plasmodium* infections in mosquitoes varying from 5 to 20% were recorded in *An. funestus* and *An. gambiae* [59].

The West and North-West regions are all situated in highland areas (> 1000 m above sea level) and characterized by a temperate climate with rainfall lasting up to 8 months and a vegetation dominated by grasslands. These areas are considered as hypoendemic with seasonal malaria parasite transmission occurring at very low level. Average annual rainfall is estimated at 1800 mm/year. The West and North-West region has each over 1.9 million inhabitants. The West region covers a surface area of 13,892 km^2^ whereas the North-West covers a surface area of 17,300 km^2^. A survey conducted in the 1990s before the scale-up of LLINs in these settings, indicated parasite prevalence reaching up to 25% in children of less than 15 years-old (*n* = 530) [44, 60]. Entomological inoculation rate in the West region was found to vary from 62.8 to 90.5 infective bites/person/year [61]. After LLINs scale-up, a parasite rate varying from 9.3 to 22.4% (*n* = 173) in febrile children of 2–15 years-old was reported in different health care units of the West region [8, 58]. Retrospective analysis conducted between 2006 and 2012, in the health care district of Mbakong (North-West), showed a decrease in the parasite rate in febrile patients from 53.2% to 18.2% (*n* = 4230) following LLINs scale-up and a usage rate of > 50% [62]. Current entomological investigations reported parasite inoculation rates varying from 4.9 to 11 infective bites/person/year in the highland areas of the North-West region [63], whereas 2.24 infective bites/person/month was recorded in the West region (Table 2) [61, 64]. Main vectors in the area are *An. gambiae*, *An. coluzzii* and *An. funestus*.

The Littoral, Centre, South-West, East and South regions all belong to the forest domain. This domain extends from the Atlantic coast to the border with the Central African Republic and is characterized by a succession of vegetation, including mangrove, deep equatorial evergreen forest and humid savannah. The climate comprises four seasons, two rainy seasons and two dry seasons with annual rainfall varying between 1500 mm/year inland to 4000 mm/year on the sea coast. These regions are considered as belonging to a holoendemic stratum with high and perennial malaria parasite transmission. The Littoral, Centre and South-West regions are the most densely populated, with the population varying from 4.09 million for the Centre, 3.3 million for the Littoral and 1.5 million inhabitants for the South-West region. The East and South regions are the less densely populated with a population of 745,000 inhabitants in the South and 832,000 inhabitants in the East region (Table 1). Prior to LLINs scale-up, the prevalence rate in children aged 6 months to 15 years-old ranged from 35 to 85.4% (*n* = 109–1690) [44, 65–69]. Studies conducted in the South-West region after LLINs mass campaigns scale-up, on children aged one month to 14 years-old, indicated a prevalence varying from 9 to 41.5% (*n* = 454) during the rainy season in Tiko, Limbé, Idenau, Mutengene and Buéa [18, 68, 70–75]. High parasite prevalence varying from 41.7 to 56.2% (*n* = 828) along the slope of Mount Cameroon was also recorded [76, 77]. The social crisis affecting the North-West and South-West regions of the country with a displaced population, could be affecting malaria epidemiology in the area and this could constitute an enormous challenge that could impede malaria elimination or control efforts in these regions and neighbouring regions. In the South and Centre regions a decrease in malaria parasite prevalence was recorded in the majority of settings with estimates of 6.6–29.5% (*n* = 2525) [58, 78, 79]. However, high parasite prevalence estimates were still recorded in some places such as Nkolbisson (43.4%) (*n* = 315) and Mfou (77.2%) (*n* = 263) [80, 81]. In the Littoral region, malaria parasite prevalence ranged between 20.4–29.4% (*n* = 288) [58, 71]. Regarding malaria parasite transmission, different patterns of transmission were reported. Before the scale-up of vector control tools, in the coastal cities of Tiko, Limbé and Ideanu, transmission estimates varied from 149 to 287 infective bites/person/year and this was similar to transmission level in the forested or highland areas (161 infective bites/person/year) [82]. Decreases in the transmission level was recorded following the scale-up of LLINs with transmission estimates as low as 0.7 infective bites/person/month in Tiko, 1.4 infective bites/person/month in Mamfe [64]. In the Littoral, Centre and South regions, transmission was reported to vary between 100 and 350 infective bites/person/year before the scale-up of LLINs [49, 83–91]. After scale-up, EIR values ranging from 0 to 100 infective bites/person/year [92–95] were reported (Table 2).

Because of the poor road state in the East region, there have not been many studies conducted in this part of the country. Yet this region is considered as one of the most affected by malaria in the country [1]. This region is also one of the most vulnerable in the country because of its proximity with the Central African Republic and receives regularly influx of population running the social crisis in Central African Republic. Because the East region could constitute a reservoir for malaria dissemination in Cameroon, it is urgent that more efforts to improve the treatment and disease prevention be undertaken to reduce the high endemicity of malaria in the area.

The cities of Yaoundé and Douala with a population of about 3 million inhabitants each [96] are similar to most of sub-Saharan cities [97, 98]. They are characterised by a rapid demographic growth, unplanned urbanization, fast development of informal settlements, large-scale practice of urban agriculture and rapid evolution of insecticide resistance in vectors [96, 99–101], which all probably affect the dynamics and epidemiology of vector-borne diseases. Before LLINs scale-up, EIR varied between 0–33 infected bites/person/year. Parasite prevalence was reported at 35% (*n* = 965) in children of 0–15 years-old [66]. The parasite rate in febrile children of less than 15 years-old admitted in hospital was equal to 42.9% (*n* = 415) [102]. After LLINs scale-up, EIR levels of 0 to 90 infective bites/person/year and a prevalence of 35% in children aged 3–14 years-old in the general population (*n* = 236) were recorded [101, 103].

## *Plasmodium* species

All four human *Plasmodium* species have been documented in Cameroon, including *P falciparum*, *P. ovle*, *P. malariae* and *P. vivax* [6, 8, 9]. *Plasmodium falciparum* is by far the predominant species recorded in up to 95% of all infection cases [104, 105]. *Plasmodium malariae* and *P. ovale* represent each 1 and 3% of infection cases, respectively [9]. However, the distribution of the different *Plasmodium* species across the country could be underestimated as suggested by recent studies using molecular tools which indicated *P. malariae* infection cases in 17% out of 236 blood samples analysed [95, 106]. The study suggested the need to associate molecular tools in diagnostics to improve species detection. Genetic structure studies of *P. falciparum* suggested high diversity of circulating strains in Cameroon [107, 108].

*Plasmodium vivax* was recently reported from Cameroon [6–8]. Studies conducted so far suggested frequent occurrence of this parasite in Duffy-negative people in different epidemiological settings [6–8]. In the city of Dschang (West Cameroon), out of 484 blood samples collected consecutively from febrile outpatients attending the main hospital during a 3-month period, *P. vivax* infection was detected by PCR in 5.6% (27/484) patients, representing 38.6% (27/70) of all *Plasmodium* infection cases detected [8]. Another study conducted in Bolifamba (South-West Cameroon) indicated that 14.9% (13/87) of *Plasmodium* infection cases were caused either exclusively or concomitantly by *P. vivax*, in individuals both positive (50%) and negative (50%) for the Duffy receptor [6]. In a larger study conducted in five locations in the South region of Cameroon, out of 201 malaria positive cases detected, six *P. vivax* and two mixed parasite infections (*P. falciparum* + *P. vivax*), were detected corresponding to a prevalence of 4% [7]. Yet the true profile of species occurrence and distribution across the country is not well documented. It is possible that *P. vivax* could have been misdiagnosed as *P. ovale* in the past since distinction in routine microscopy is very difficult. In neighbouring Equatorial Guinea, cases of *P vivax* infection are also highly prevalent [109, 110]. It is thought that the influx of workers from countries where *P. vivax* is endemic (Indonesia, Philippines) following the discovery of oil reserves in Equatorial Guinea could have increased *P. vivax* reservoir and transmission [110]. It is not clear whether subsequent expansion of the parasite in Cameroon could have resulted from population migration between the two countries. The discovery of *P. vivax* in Cameroon offers new research avenues on this species distribution, pathogenicity, genetic variability, transmission by different vectors species, interaction with other *Plasmodium* species and distribution in regard to urbanisation, climatic changes or malaria treatment policy. The expansion of *P. vivax* in Cameroon could oppose important challenges for malaria elimination in the country. One of these challenges could be the adoption of primaquine for radical cure of *P. vivax* related cases. *Plasmodium vivax* infections differ from other *Plasmodium* species because the parasites can lie dormant in a person’s liver, and reawaken suddenly later to cause relapses of malaria. Primaquine is thus taken daily for 14 days to clear *P. vivax* parasites in the blood and liver to prevent subsequent relapses. However, this drug is considered to be associated to serious adverse effects (destruction of red blood cells), particularly for patients with hereditary deficiency of the enzyme glucose-6-phosphate dehydrogenase (G6PD) [111–113] and might require specific case management strategies. Another challenge is the proper diagnostic and procurement of new RDT tests for the detection of *P. vivax* infections in patients.

Great apes in Cameroon were also reported to harbour several *Plasmodium* species close to *P. falciparum*, *P. vivax*, *P. malariae* and *P. ovale* [114–116]. Six *Plasmodium* species closely related to human-infecting *P. falciparum* were reported in the central African equatorial forest region. These include *P. reichnowi*, *P. gaboni* and *P. billcollinsi* found in chimpanzees, and *P. adleri*, *P. blacklocki* and *P. praefalciparum* found in gorillas [117]. Anopheline species such as *An. moucheti* was considered to be a possible bridge vector between human and apes [118]. *Plasmodium falciparum*-like parasites infecting wild apes in southern Cameroon were not found to represent a recurrent source for human malaria [106]. In Malaysia, Southeast Asia, recurrent cases of human infections by *Plasmodium knowlesi*, an Asian simian malaria parasite, were regularly reported [119]. Although subsequent malaria control interventions induced a marked reduction in the incidence of *P. falciparum* and *P. vivax* malaria cases, an increase in the incidence of malaria cases from the simian parasite *P. knowlesi* was still recorded [120–123]. Given the potential for simian *Plasmodium* to be transmitted from human to human and the public health implications of this zoonosis, it becomes important that more surveillance activities be conducted on this end through frequent diagnostic of simian *Plasmodium* in blood donors or resident living close to primates in the forest regions. It is still not known whether primates could represent a reservoir for human-infecting *Plasmodium*. Moreover, in the context of malaria elimination, identifying sources for reinfection of mosquitoes or possibilities of parasite introgression could have implications for the successful implementation of vector control programmes.

## Malaria treatment and case management

Following expansion of drug resistance, drug policy for malaria treatment in Cameroon gradually changed over the years from monotherapies with chloroquine and amodiaquine used as a first-line treatment for uncomplicated malaria to combination therapy [124]. Chloroquine was largely used from the 1970s through to 2002 [9]. From 1999 to 2004, following the adoption of an interim drug policy, amodiaquine was incorporated alongside chloroquine as an alternative first-line drug for uncomplicated malaria while sulfadoxine-pyrimethamine was used as a second line drug [9, 124]. In 2004, following recurrent treatment failure to amodiaquine and sulfadoxine-pyrimethamine, the Ministry of Health of Cameroon reconsidered its policy and shifted to artemisinin-based combination therapy (ACT) used as a first-line treatment for uncomplicated malaria. Common ACT used in the country include artesunate-lumefanthrine, artesunate-atovaquone-proguanyl, artesunate-amodiaquine, artesunate-mefloquine. Sulfadoxine-pyrimethamine is still recommended as an intermittent preventive treatment for malaria during pregnancy (IPTp) whereas injectable artemether or quinine are used in case of treatment failure or for severe malaria cases [9]. In the northern part of the country exposed to recurrent malaria outbreaks during the rainy season, the government introduced in 2016 seasonal malaria chemoprevention for children below 5 years-old [1, 14]. The combination artesunate-amodiaquine (ASAQ) which was used before for the treatment of uncomplicated malaria cases for children under 5 years-old was replaced by artemether-lumefantrine (AL) provided free of charge to all families for malaria prevention. This strategy permitted to take in charge over 80% of children in the target settings in the North and Far-North regions [1].

Case management in Cameroon includes: diagnosis of suspected cases; treatment of confirmed cases at health facilities and community level; scale-up of integrated community case management; pharmacovigilance and supply chain strengthening. Since 2011 the Cameroon government adopted free treatment of uncomplicated malaria for children of less than five years-old [1, 14]. Malaria diagnosis in most health care units is done through microscopic and/or TDR [1, 14, 20]. Since 2014 treatment of severe malaria is also free for children under 5 years-old [1, 14]. Integrated community case management (iCCM) for diarrhoea, pneumonia and malaria using community health workers, was introduced in 2009 to target groups with difficult access to health care services [125]. ICCM include clinical diagnosis and treatment provided by trained and supervised community health workers (CHWs). The results of pilot programmes conducted in the East region of Cameroon (Doume and Nguelemendouka) with 456 trained community health workers indicated that this approach improve equitable access to treatment for malaria and diarrhoea in remote settings of Cameroon [125]. The implementation of modified iCCM programme with proactive screening of children of < 5years-old in high malaria transmission settings (Bare Bakem in the Littoral region) showed that it could increase the likelihood to find malaria parasite infections in children by > 67% [126]. In different settings where iCCM have been introduced it is reported to have increased the treatment rate for malaria, care seeking behaviour for fever, and has reduced the burden on health care facilities [127]. Yet this approach faces several challenges such as underutilisation or the attrition of trained CHWs, inadequate supervision and motivation of CHWs, prolonged and frequent unavailability of commodities for malaria diagnosis and treatment [126]. Concerning pharmacovigilance several studies have so far been conducted across the country to assess malaria drug efficacy. The efficacy and safety of artemisinin base combination have been evaluated in four sentinel sites, Garoua, Bamenda, Nkongsamba and Ebolowa [128, 129]. Also, eight generic artemisinin base combinations have been evaluated from 2005 to 2016 [24, 103, 124, 129, 130]. All these studies concluded to the continuous efficacy of artemisinin base combination in Cameroon [128, 129, 131]. For diagnostic and treatment of malaria cases, several programmes have been conducted across the country in order to strengthen health care workers practices [132–134]. Additional case management programmes conducted include seasonal malaria chemoprevention in the North and Far-North regions.

In order to achieve disease elimination, proper detection and treatment of malaria cases is required and the contribution of case management could become even more determinant, particularly in settings selected for malaria elimination, where the objective will be to track all cases to avoid reintroduction of malaria infected patients in malaria free zones.

## Drug resistance

Current therapeutic efficacy studies suggest continuous efficacy of artemisinin-based combinations (with complete parasite clearance on day 3) in the country despite slight decline from 97% in 2006 to 90.2% in 2016 for artesunate-amodiaquine (AS-AQ), a compound largely used for malaria treatment in Cameroon [14]. Although these values are still largely in favour of a high efficacy of this combination there is a need to remain vigilant to avoid rapid expansion of drug resistance which could threaten the successful elimination of malaria. Yet if AS-AQ continues to be used as the official first-line treatment, then a policy change would need to be considered in the years to come. Fortunately, the Ministry of Health has started adopting artemether-lumefantrine as a first-line treatment in some part of the country [1, 14]. In Cameroon as in most countries in sub-Saharan Africa, almost half of drugs sold on the market or in some private health care units are fake and counterfeit medication of low quality [135]. Over 50% of the population get recourse to these drugs for their treatment [136, 137]. It is considered that substandard or fake antimalarials cause the death of 64,000 to 158,000 people in Africa in the recent years [138]. Controlling the quality of drugs sold on the market for improved case management constitute an important requirement to further consider in the perspective of malaria elimination.

Drug efficacy has been reported to be affected by mutations occurring in the *Pfcrt* and *Pfmdr1* gene in *P. falciparum* [139–142]. Mutations in the *Pfcrt* gene in *P. falciparum* are known to be associated with chloroquine and amodiaquine resistance [140, 143], whereas mutations in the *P. falciparum* multidrug resistance 1 (*Pfmdr1*) are considered to confer resistance to a large set of compounds including chloroquine, mefloquine, halofanthrine and quinine [142, 144, 145]. *Pfdhfr* and *Pfdhps* alleles are considered to mediate resistance to sulfadoxine-pyrimethamine [146, 147]. Studies conducted between 2005 and 2009 indicated a high prevalence of *Pfcrt* 76T mutation in various sites across Cameroon [148, 149]. The *Pfmdr1* 86Y mutation was also recorded at high frequency [150] however, no mutations in the *Pfcrt* 72 and no duplication of the *Pfmdr1* gene were detected [149, 151, 152]. A recent study assessing the evolution of resistance genes in *P. falciparum* in the South-West region of Cameroon in blood samples collected between 2003 and 2013, indicated rapid elimination of alleles conferring resistance to 4-aminoquinoline (chloroquine and amodiaquine) *Pfcrt* 76T, *Pfmdr1*, 86Y, 184F and 1246Y and return to chloroquine sensitive genotypes since the withdrawal of chloroquine [20, 153]. However different evolutionary patterns of mutations associated with *Pfcrt* gene have been reported across the country, with novel mutations still reported from different settings, notably the Centre and South regions [148, 154]. For *pfdhfr/pfdhps* genes, no reduction in SNPs associated with antifolate drug resistance was recorded [20]. A study on pregnant women in the city of Yaoundé confirmed the presence of an increasing number of mutations on the *Pfdhfr/Pfdhps* genes [147]. Apinjoh et al. [72], described the presence of triple mutants on the *Pfdhfr*, *Pfcrt*, *Pfdhps* and *Pfmdr1* genes in the South-West region (Table 3). The increase in the prevalence of mutations could result from intense selective pressure still going on with the use of sulfadoxine-pyrimethamine for chemoprophylaxis by pregnant women and other vulnerable groups. For the *Pfkelch* 13 gene few random mutations have been recorded. However, none of the mutations associated with artemisinin resistance in Southeast Asia have so far been recorded [72, 155, 156]. Yet Cameroon remains extremely vulnerable to potential risk of introduction and spread of artemisinin resistant mutations originating from Southeast Asia with the United Nation peace keeping operations with soldiers from Asia (Bangladesh and Pakistan troops) in Central African Republic [157] or oils workers from Asia in the neighbouring Equatorial Guinea [110]. The following stresses the need for regular surveillance activities to avoid the rapid spreading of these new mutations in Cameroon and the sub-region. It should also be important to conduct regular monitoring of the therapeutic efficacy of artemether-lumefantrine now widely used across the country.

**Table 3:** Most prevalent drug resistant mutations in *Plasmodium falciparum* reported during recent years across Cameroon

Gene	Drugs affected by gene mutations	Mutations detected in Cameroon	Sites reporting drug resistance	References
*pfcrt*	CQ, AQ, LM	K76T, M74I, N75E, K76 T, Q271K, I356K	Mutengene, Ebolowa, Yaoundé, Bertoua, Douala, Kyé-Ossi, Ngaoundéré, Garoua	[7, 17, 20, 147–149]
*pfdhfr*	SP	N511, C59R, S108N	Yaoundé, Mont-Cameroon, Ngaoundéré, Garoua	[17, 20, 147–149]
*pfdhps*	SP	K142N, I431V, S436A, A581G, A613S, K540E, A437G	Yaoundé, Mont-Cameroon, Mutengene, Garoua	[17, 20, 147, 149]
*pfmdr-1*	AQ, CQ, LM, MQ	N86Y, Y184F, D1246Y	Mutengene, Yaoundé, Garoua	[17, 20, 149]
*pfkelch13*	Artemisinine	K189T^a^	Mont-Cameroon	[17]

^a^This mutation does not confer resistance to artemisinine

*Abbreviations*: *pfdhfr*, *P. falciparum* dihydrofolate reductase; *pfdhps*, *P. falciparum* dihydropteroate synthase; *pfcrt*, *P. falciparum* chloroquine resistance transporter; *pfmdr-1*, *P. falciparum* multidrug resistance transporter 1; CQ, chloroquine; AQ, amodiaquine; LM, lumefantrine; SP, sulfadoxine-pyrimethamine; MQ, mefloquine

## Vectors species distribution, bionomics and genetic variability

Cameroon has one of the most diverse anopheline fauna in Africa with more than 50 species reported [10]. Sixteen of the species are recognised as main or secondary malaria vectors and are involved in malaria parasite transmission either permanently or occasionally [11, 53, 63]. Species considered as main malaria vectors include: *An. gambiae* (*s.s.*), *An. coluzzii*, *An. arabiensis*, *An. funestus*, *An. nili* and *An. moucheti* (Table 2). Recent progress in molecular biology and genomics has allowed in-depth studies on species distribution, bionomics, genetic variability and geographical distribution across the country. The distribution of these species is now well documented in favour of intensive field studies undertaken across the country [11, 158–161]. Secondary malaria vectors include species which are involved in malaria parasite transmission either occasionally or temporally. Up to 11 species have been classified in this group which comprises: *An. ovengensis*, *An. paludis*, *An. ziemanni*, *An. coustani*, *An. pharoensis*, *An. marshallii*, *An. rufipes*, *An. carnevalei*, *An. hancocki*, *An. leesoni* and *An. wellcomei* [11, 52, 53, 63] (Table 4).

**Table 4:** Characteristics of species groups involved in malaria parasite transmission in Cameroon

Species complex/group	No. of species in the group	Present in Cameroon	Resting behaviour	Feeding behaviour	Role in malaria parasite transmission in Cameroon	Geographical distribution
*An. gambiae* complex	9	*An. gambiae*	Endo/exophilic	Anthropophilic	High	Countrywide
		*An. coluzzii*	Endo/exophilic	Anthropophilic	High	Countrywide
		*An. arabiensis*	Exophilic	Anthropozoophilic	High	Sahelian and savannah
		*An. melas*	Exophilic	Anthropophilic	Unknown	Coastal
*An. funestus* group	11	*An. funestus*	endophilic	Anthropophilic	High	Countrywide
		*An. leesoni*	Exophilic	Anthropophilic	Minor	Countrywide
		*An. rivulorum*	Exophilic	Zoophilic	Unknown	Dry savannah
		*An. rivulorum-like*	Exophilic	Zoophilic	Unknown	Dry savannah
*An. nili* group	4	*An. nili*	Exophilic	Anthropozoophilic	High	Countrywide
		*An. ovengensis*	Exophilic	Anthropozoophilic	Moderate	Forest
		*An. carnevalei*	Exophilic	More zoophilic	Minor	Forest
		*An. somalicus*	Exophilic	Zoophilic	None	Forest
*An. moucheti* group	3	*An. moucheti*	Endophilic/ exophilic	Anthropophilic	High	Forest
*An. coustani* group	8	*An. coustani*	Exophilic	More zoophilic	Moderate	Countrywide
		*An. ziemanni*	Exophilic	More zoophilic	Moderate	Countrywide
		*An. paludis*	Exophilic	Anthropophilic	Moderate	Forest
		*An. namibiensis*	Exophilic	Anthropophilic	Unknown	Forest
		*An. obscurus*	Exophilic	Zoophilic	Unknown	Forest
*An. marshalli-hancocki* group	12	*An. marshallii*	Exophilic	More zoophilic	Minor	Forest
		*An. hancocki*	Exophilic	Anthropophilic	Minor	Forest
		*An. brohieri*	Exophilic	Zoophilic	Unknown	Savannah
		*An. wellcomei*	Exophilic	More zoophilic	Minor	Forest
		*An. njombiensis*	Exophilic	Zoophilic	None	Forest
		*An. hargreavsi*	Exophilic	Zoophilic	None	Forest
*An. pharoensis*	1	*An. pharoensis*	Exophilic	Anthropozoophilic	Moderate	Sahelian and savannah
*An. rufipes* group	2	*An. rufipes*	Exophilic	Anthropozoophilic	Moderate	Sahelian and savannah

### *Anopheles gambiae* complex

Members of the *Anopheles gambiae* species complex found in Cameroon include *An. gambiae* (*s.s.*), *An. arabiensis*, *An. coluzzii* and *An. melas* [158, 161]. While *Anopheles arabiensis* is restricted to the northern arid and semi-arid zone, *An. gambiae* (*s.s.*) and *An. coluzzii* are widely distributed across the country [11, 158]. *Anopheles gambiae* (*s.s.*) and *An. coluzzii* are highly anthropophylic and closely related to anthropogenic environments for resting and oviposition [162, 163]. Yet recent findings in different ecological settings suggested high phenotypic plasticity of feeding, biting or resting behaviours for *An. gambiae* (*s.s.*) and *An. coluzzi* with increased usage of protection measures such as LLINs [12, 164–166]. It is likely that this might reflect a shift in the feeding or resting behaviour of these species or could just be the suppression of most vulnerable taxa by the use of indoor based interventions. More malaria parasite transmission cases occurring outdoors have been reported in different epidemiological settings [101], thus suggesting the need for interventions targeting outdoor biting mosquitoes such as spatial repellents or larval control [167]. Similar strategy would apply to *An. arabiensis* which is exophagic and exophilic and feeds on both humans and cattle [53]. This species behavior has not been deeply affected by the implementation of control measures across the country, probably because of the high number of people frequently sleeping outdoor part of the year due to heat and hot temperature in the northern part of the country where this species predominates [53]. Rapid expansion of insecticide resistance as a result of increased use of LLINs and pesticides in agriculture has been reported in this species [168, 169]. *Anopheles coluzzii*, *An. gambiae* (*s.s.*) and *An. arabienis* are frequently involved in malaria parasite transmission in Cameroon and sometimes in sympatry, with infection rates varying from 2 to 10%, and entomological inoculation rate (EIR) reaching up to 400 infective bites per person per year depending on the epidemiological setting [11, 52, 53]. By contrast, there is still no record on *An. melas* implication in malaria parasite transmission in Cameroon, but this species is considered as a good vector in the neighbouring Equatorial Guinea [170]. Further comparative studies are required between *An. melas* populations from Cameroon and Equatorial Guinea to understand their differences in vectorial capacity.

Although *An. gambiae* (*s.s.*) and *An. coluzzii* overlap to a large extent, species distribution modelling studies revealed differences in the ecological niche of the two species [161]. *Anopheles coluzzii* and *An. gambiae* (*s.s.*), segregate along two gradients: distance from the coastline and altitude, with *An. coluzzii* displaying a bimodal distribution, predominating in dryer savannah and along the western coastal fringe [171]. Recent evolutionary studies testing the relationship between reproductive isolation, ecological divergence and hybrid viability suggested a positive association between the strength of reproductive isolation and the degree of ecological divergence. These findings indicate that post-mating isolation does contribute to reproductive isolation between these species [172]. At micro-environmental level, studies conducted in the city of Yaoundé indicated that *An. gambiae* (*s.s.*) and *An. coluzzii* could segregate along the urbanization gradient with *An. coluzzii* being more adapted to urban settings and *An. gambiae* (*s.s.*) in rural settings [173]. The current speciation between *An. coluzzii* and *An. gambiae* (*s.s.*) and their adaptation to different type of habitats in the urban environment including polluted sites, artificial containers and possible changes in their vectorial competency [99, 100, 174] warrants further investigations. In Cameroon according to the latest population census, over 52% of the population live in urban settings and this population is projected to grow faster in the next decades [40]. Studies conducted so far in the main cities of Cameroon (Yaoundé and Douala) suggested that unplanned urbanization and the practice of urban agriculture maintain high malaria transmission risk by providing suitable habitats for mosquitoes [93, 99, 101, 175]. If no measures are taken, this could lead to more malaria cases in urban settings and high incidence of severe malaria in both adults and children since people living in urban settings have less premunition against malaria [176–178].

### *Anopheles funestus* group

*Anopheles funestus* is a group of 11 species distributed across Africa. In Cameroon four species have been reported including *An. funestus*, *An. leesoni*, *An. rivulorum* and *An. rivulorum*-like which differ from the type-form by slight genetic differences [179]. Although this variant has been reported from Cameroon, Burkina Faso and South Africa, its taxonomic status and role as vector are still unclear and warrant further investigations in order to know if it could be a target for vector control interventions [179, 180]. Yet a modified version of the species complex PCR originally set up by Koekemoer et al. [181] was designed by Cohuet et al. [179] to enable identification of this variant. Within the members of the group present in Cameroon only *An. funestus* and *An. leesoni* have so far been reported infected [11, 160]. *Anopheles funestus* is highly endophilic and feed predominantly on humans [182, 183]. This species was responsible for infection rate of up to 10% and EIR of up to 350 infected bites per person per year [11, 91, 184]. Genetic analysis conducted on *An. funestus* using microsatellite markers reported high panmixia between vector populations and a genetic differentiation of populations consistent with isolation by distance [185]. Cytogenetic studies and fine-scale mapping studies, demonstrated high level of chromosomal heterogeneity both within and between populations, which could reflect the influence of both ecotypic variations and environmental factors [185–188]. Although there have been several studies exploring the bionomics, susceptibility to insecticides, resistance mechanisms over recent years [57, 59, 95, 189–197], the influence of the intensification of control measures on the species bionomics, genetic structure and vectorial capacity is not well understood and the following could impede the successful completion of disease elimination programmes to be conducted in Cameroon.

### *Anopheles nili* group

This group consists of four species including *An. nili* (*s.s.*) (the type-form), *An. carnevalei*, *An. ovengensis* and *An. somalicus* [10, 198, 199]. *Anopheles nili* is by far the most important vector species of the group. *Anopheles ovengensis* and *An. carnevalei* have been found infected in Cameroon [11, 90], while *An. somalicus* is strictly zoophilic and is not therefore involved in malaria parasite transmission [90, 199]. *Anopheles nili* and *An. ovengensis* bite indoors as well as outdoors but rest mainly outdoors. *Anopheles carnevalei* bites exclusively outdoors [90, 199]. There has been substantial progress in the recent years regarding genetic studies on *An. nili*. These studies include the development and chromosomal mapping of microsatellites makers, development of chromosome maps for *An. nili*, *An. ovengensis* and *An. carnevalei*, and development of single nucleotide polymorphisms (SNPs) for fine scale genomic analysis [200–203]. Genetic structure analysis conducted for members of the *An. nili* group using microsatellites, sequencing of ribosomal DNA and fine-scale mapping indicated high level of genetic differentiation between these species [203, 204]. Studies conducted on the type-form *An. nili* (*s.s.*) suggested high gene flow between populations situated across the distributional range of the species in West and Central Africa [205]. However, a cryptic genetic diversity within *An. nili* (*s.s.*) was reported in the deep equatorial forest environment of South Cameroon, reflecting a complex demographic history for this major malaria vector in this environment [204]. Cytogenetic analysis indicated the occurrence of two chromosomal inversions displaying high frequencies in the savannah compared to the forest populations that are purported to be related to local selection or adaptation to climatic cline [201]. *Anopheles nili* is highly predominant in villages near permanent rivers which constitute it breeding sites and can easily be localized and targeted for vector control, but due to their high outdoor feeding and resting behaviour, members of the *An. nili* group are less affected by indoor based interventions. Also, their implications as bridge vector for primate-infecting *Plasmodium* spp. as well as for other wild parasites or virus need to be evaluated. Such information could be crucial in the perspective of malaria elimination in central and West Africa where mosquitoes of the *An. nili* group play important roles in malaria parasite transmission.

### *Anopheles moucheti* group

This group consists of three sub-species namely *An. moucheti moucheti* (hereafter *An. moucheti*) (the type-form), *An. moucheti nigeriensis* and *An. moucheti bervoetsi*. These subspecies could be distinguished from one another by slight morphological characters or by the use of a PCR molecular assay [206–208]. In Cameroon, only the type-form of *An. moucheti* is present, and is mainly found in the equatorial forest domain where it has been reported to be responsible for transmission rates reaching 300 infective bites/person/year, particularly in villages situated along slow moving rivers [11, 88, 199, 209]. Genetic studies conducted on this mosquito population indicated high genetic variability and low genetic differentiation between populations distributed across the range of the species in Cameroon, the Democratic Republic of Congo and Uganda [210, 211]. Recent studies allowed the development of a chromosomal map for the species [212]. In the light of studies conducted so far in Gabon [118], it is not clear whether *An. moucheti* populations in Cameroon are competent for the transmission of *Plasmodium* spp. infecting the great apes. Because of the circulation of primate-infecting *Plasmodium* in the equatorial forest region, it could be interesting to run experimental infection assay with *An. moucheti* to determine its competence for these primate-infecting *Plasmodium* species and for other haemoparasites found in the wild. This information could be determinant for identifying sources for zoonosis infections or those in circulation in the forest regions. More recently, studies conducted in the equatorial forest region indicated a change in the biting and resting behaviour from indoor to outdoor of this species following the intensification of vector control measures in Cameroon [164]. The implication of these findings on the performance of control measures need to be assessed in various sites and new strategies to mitigate the impact of outdoor and residual transmission developed.

## Vector control in Cameroon

In the 1940s during the colonial period, mosquito control was conducted by hygiene and sanitation services in the two main cities of Cameroon, Douala and Yaoundé [213]. Control interventions during that period were mainly based on the strict policy of regular inspection and destruction of all temporary larval habitats near houses, elimination of garbage near houses and clearing of bushes. Inhabitants from the two cities were instructed to keep their nearby environment clean and if this was not done, they could be sent to jail [213, 214]. For permanent water collections, the following compounds were used as insecticides: formol, pyrethre powder, tobacco smoke, phenic acid, quinoleine and cresyl [213, 215]. From 1949, larval control operations and house spraying were undertaken regularly by hygiene services in both Douala and Yaoundé to stop malaria parasite transmission [216]. These measures were later replaced by the launching of malaria eradication campaigns which began in 1953 using indoor residual spraying (IRS) with DDT, dieldrin and HCH as the main insecticides [216–218]. These IRS campaigns which were initiated by the World Health Organization (WHO) were replicated in different countries across sub-Saharan Africa in Senegal, Burkina Faso, Liberia, Benin and Tanzania [218]. Pilot IRS campaigns in Cameroon were conducted in Yaoundé and the northern city of Maroua and surrounding communities covering a total population of 750,000 and 250,000 inhabitants respectively. Campaigns in Yaoundé and its surrounding areas were conducted from 1953 to 1960 and was divided in two large areas; the western zone sprayed using DDT while the eastern zone was treated using dieldrin [216, 218]. These campaigns resulted in a significant decrease in malaria parasite transmission, vector density and the incidence of malaria cases to close to zero [216], but the programme was interrupted in the 1960s due mainly to financial constraints. In the northern part of the country, Maroua and its surroundings, IRS campaigns were undertaken from 1953 to 1961 using DDT only. In 1959, DDT resistance was reported in *An. gambiae* populations [217, 219]. By contrast to the control programme initiated in Yaoundé, no reduction in vector density, malaria parasite transmission nor of the incidence of cases was reported [217]. Other factors which contributed to the poor performance of the programme were the poor residual effect of the insecticide on different housing material, the inaccessibility of some villages during the rainy season or the exophilic and opportunistic behaviour of the main vector in the area, *An. arabiensis*, whereas in Yaoundé vector populations were found to be highly endophilic [217]. The programme was later stopped in 1961 due to it poor performance. Similarly to Cameroon, the global malaria eradication campaigns piloted by the WHO across Africa also failed. As a consequence, the WHO initiated the Garki Malaria Project in Kano, Nigeria [220]. This pilot project intended to better appraise the epidemiology of malaria and assess whether malaria elimination could be achievable in West African savanna area with high and perennial malaria parasite transmission using mass drug administration and IRS [220]. Although the project was well thought, it also failed despite lessons learn from previous campaigns and the use of a different strategy associating mathematical modelling analysis, intensive application of propoxur in houses and widespread distribution of the drugs chloroquine and sulfadoxine-pyrimethamine to the population [220]. The prevalence of malaria only dropped from 80% to 30% after a three-year campaign and returned to 80% one year after the stoppage of the programme [220]. The authors of the study indicated that high entomological inoculation rate due to principal vectors and the complexity of malaria ecology in the area, were the reasons of the programme failure. Yet the Garki Project provided a high number of findings which are relevant nowadays and for future control operations [221]. The failure of the global malaria eradication campaign and the very limited impact of the Garki Project interventions throw light on the heterogeneous epidemiology of malaria across Africa and the need for a better understanding of the factors affecting disease transmission.

Following the unsustained results of malaria pre-eradication and eradication campaigns across sub-Saharan Africa [222, 223], the WHO adopted a change of strategy from vector control to prioritizing treatment and chemoprophylaxis [224]. Cameroon also aligned its policy on this same direction. The massive use of chloroquine during the 1960s through to the 1990s was associated with the appearance of resistance, which spread widely across the continent [225]. In Cameroon, the first cases of chloroquine resistance were recorded in the early 1980s [124, 226–228].

A large-scale vector control programme was resumed in the country in the 1990s with the deployment of pyrethroid-treated nets. Several pilot programmes were conducted across Cameroon. Trials conducted in Edéa, Mbébé, Ebogo, Kumba and Mbandjock in forest and humid savannah areas against vector species such as *An. gambiae* (*s.l.*), *An. funestus*, *An. nili* and *An. moucheti*, provided sufficient evidence for the scale-up of this intervention across the country [83, 89, 229–231]. At the level of the Ministry of Health, key actions were undertaken to speed up the scale-up of treated nets across the country to prevent malaria. This included: (i) the development of a malaria control strategic plan with the goal of achieving 60% coverage of the target population by 2006; (ii) the creation and equipment of 10 reference units for bed-net impregnation in the ten regions of the country; (iii) the training of local staff to undertake bed-net impregnation; (iv) the organisation of free distribution campaigns of bed-nets to pregnant women and children of under five years-old; and (v) the inclusion of up to 1733 NGO and local community groups in the promotion of the use of treated nets. These actions permitted to attain a coverage rate which gradually increased from 5.7% in 2003, 16.6% in 2004 and 39.5% in 2005, with yet a high variability of the level of coverage between regions noted [232].

Nowadays, malaria prevention in Cameroon mainly relies on the use of long-lasting insecticidal nets (LLINs) (of different brands, e.g. PermaNet, Olyset, Interceptor) [21, 233, 234]. Since 2004 three important free distributions of treated nets have been conducted across the country. The first in 2004–2005 permitted the distribution of up to 2 million insecticide treated nets to pregnant women and children younger than 5 years of age. The second campaign conducted in 2011 allowed the switch from ITN to LLIN with the distribution of up to 8 million LLINs to the general population, while the third in 2015 permitted the distribution of over 12 million LLINs to the entire population [1, 235]. It is estimated that 77% of the population own at least a treated net and that 58% of the population use these nets regularly [1]. Yet heterogeneous patterns between LLINs ownership and utilization have been reported in different epidemiological settings across the country [133, 137, 236–239]; this is considered as an important factor affecting the performance of treated bed-nets in Cameroon. Although several sensitization campaigns through the media, or using community workers or through meetings with the communities have been conducted across the country to increase bed-net usage [236] it remains low. Disparities in bed-net ownership and usage between regions in Cameroon could be linked to cultural, social practices or lifestyle [240]. It is becoming urgent to involve more social science specialists in vector control interventions in order to address population adherence to these interventions. Since the introduction of LLINs, significant reduction of both entomological and epidemiological indicators has been documented across the country by different studies [24, 199] thus stressing the importance of this tool for malaria control in the country. From national statistics it appears that some regions display high coverage or usage rate of LLINs compared to others; however, these same regions, equally display high estimates of malaria prevalence and transmission [1]. This contradicting figure derives from the fact that the true usage or coverage rate could be underestimated since self-reports through questionnaires are usually used to collect information from households [24]. In a recent study in the city of Yaoundé, it was reported that self-reporting overestimated by 10–30% the average usage rate of bed-net by the population compared to the national level [136]. Self-reported measures have been found to overestimate ITN adherence by over 13% elsewhere [241]. It is becoming important that, different methods be used to collect information from households to assess coverage and utilization of LLINs. In Zambia, mass distribution of LLNs through door-to-door delivery to households in rural settings associated with net hanging and face-to-face health education on LLIN use and ways of reducing net wear and tear were found to increase the usage and coverage rates [242]. If correctly used and high coverage rate achieved, LLINs could have a central role in the path to achieving malaria elimination in the country. It should be interesting to preserve LLINs efficacy by assessing the actual sustainability of the use of LLINs, practices leading to less utilization of nets after a certain time, the quality of nets delivered to the population, the persistence of active ingredient on nets and the efficacy of LLINs at different periods.

In addition to LLINs, pilot vector control trials (larviciding and indoor residual spraying) have been launched in the country [14, 16]. The larviciding trial is undertaken in the city of Yaoundé by the team of OCEAC in collaboration with the NMCP. This programme intends to assess the efficacy of larviciding using a combination of *Bacillus thuringiensis israelensis* and *B. sphaericus* for controlling malaria parasite transmission and mitigating the impact of insecticide resistance. This study intends to provide critical information which could be useful for adopting larviciding as a complementary approach for controlling malaria parasite transmission in major cities in Cameroon [167]. The second programme conducted by the VectorLink project under the sponsorship of the US President Malaria Initiative (PMI), is indoor residual spraying. This programme intends to assess the impact of indoor residual spraying for eliminating malaria in eligible sentinel sites in the two northern regions of the country [14]. The programme, which is at its initial phase, intends to provide critical information for the scale-up of similar interventions in eligible sites across the country. The city of Yaoundé is also benefiting from the PADY programme which mainly focus on hygiene and sanitation through the construction of drains on the bed of main rivers crossing the city in order to reduce permanent breeding opportunities for mosquitoes [15, 16]. However, the deployment of these tools should be accompanied by stringent routine entomological and epidemiological surveillance activities to monitor shift in incidence of cases, mosquito biting rate, entomological inoculation rate and insecticide resistance and operational issues well described and how they are solved to avoid the same problems to other teams.

## Insecticide resistance

The increased use of treated bednets and the use of insecticides in agriculture are all considered to select for insecticide resistance in mosquito populations [167]. Insecticide resistance is recognized as a serious threat for control interventions implemented in the country. The first cases of insecticide resistance in Cameroon were reported in the 1950s during malaria eradication pilot campaigns with *An. gambiae* (*s.l.*) populations exhibiting resistance to both dieldrin and DDT [218]. A recent review of data from the 1990s to 2017 indicated rapid expansion of insecticide resistance particularly to pyrethroids and DDT in the main malaria vectors *An. gambiae*, *An. coluzzii*, *An. arabiensis* and *An. funestus* across the country [167]. During the last decade important variations in the level of susceptibility of *An. gambiae* vector populations to insecticides have been documented [169, 243–246] affecting the efficacy of LLINs [247, 248]. However, a randomized control trial study conducted between 2013 and 2015 in 38 clusters in the northern part of the country where *An. arabiensis*, *An. gambiae*, *An. coluzzii* and *An. funestus* are present, suggested no influence of insecticide resistance on LLINs efficacy in preventing against malaria parasite transmission [51]. Pyrethroid resistance was found to be conferred by *kdr* West and East alleles and metabolic-based mechanisms [99, 249, 250]. Main genes reported to be involved in DDT and pyrethroid resistance in both *An. gambiae* and *An. coluzzii* include *cyp6p3*, *cyp6m2*, *cyp6p4*, *cyp9k1*, *gstd1-6 cyp6z3* and *gstd1-4*. There is still a paucity of data on the distribution of main candidate detoxification genes and of the intensity of resistance in different ecological settings. The primary mechanism conferring resistance to DDT and pyrethroids in *An. arabiensis* is mainly a metabolic detoxification mechanism. However, over recent years, an increase in the prevalence of target-site resistance, *kdr* 1014F and 1014S alleles was also reported for this species [92, 167, 251]. Despite widespread distribution of *kdr* resistance alleles in *An. gambiae* (*s.l.*) populations, *kdr* tend to be less likely than metabolic resistance to induce control failure [252].

Although less common, resistance to bendiocarb has also been reported [99, 250]. This resistance was not associated to the presence of the ACE 1 target-site mutation but is likely mediated through metabolic mechanisms [250]. Yet the presence of the ACE 1 mutation is suspected in the country [253]. This probably discards carbamates as an alternative to pyrethroids for vector control in Cameroon whereas organophosphates, which are still largely efficient, could be indicated for future vector control interventions.

With regard to *An. funestus*, several studies reported increased prevalence of insecticide resistance in this vector in both forest and savannah areas [59, 95, 194]. This resistance is mainly mediated by metabolic-based mechanisms since no *kdr* have been found in this species. The following mechanisms were reported to induce resistance: 119F-GSTe2 was found to confer resistance to DDT and pyrethroids; 296S-RDL mutation was associated to resistance to dieldrin; and several P450 monooxygenase genes were reported to be involved in resistance to both DDT and pyrethroids [189, 194]. The resistance gene 119F-GSTe2 was reported to influence life traits of both adults and larval stages of *An. funestus* [254].

With the continuous expansion of insecticide resistance in vector populations, the global programme for insecticide resistance management recommends [252] the implementation of measures to maintain the efficacy and the lifespan of current and future malaria control tools as a long-term goal. Short-term objectives are to preserve the susceptibility of major malaria vectors to pyrethroids and other classes of insecticides until new insecticides become available by using integrated control approaches which combine different interventions or tools or rotation of interventions at different periods.

## Conclusions

The present review provides an update of the situation of malaria, vectors bionomics, *Plasmodium* species distribution, case management, drug resistance, disease prevalence and control measures in Cameroon. Although significant progress has been made over the last decade to curb the disease burden, malaria is still largely prevalent across the country and displays a high complexity and heterogeneity. As the review highlighted, several challenges affect both the treatment, case management, operational implementation and vector control interventions and warrant further consideration. Malaria treatment is affected by the decrease in drug efficacy and rapid spread of resistance in *P. falciparum* populations to sulfadoxine-pyrimethamine and 4-aminoquinoline. Although artemisinin is not yet affected, there is a need to remain vigilant with the emergence of artemisinin resistance in Southeast Asia which can spread to Africa through migrants or independently emerge. In this regard, recent molecular tools could be determinant for tracking resistant genes and control failures. On the vector side, the rapid emergence of insecticide resistance that is affecting almost all compounds used in public health is a major threat for current malaria vector control programmes. In addition to core interventions (LLINs, and IRS), which mainly rely on insecticides, additional control tools such as spatial repellents, larval source management, new generation LLINs, durable wall lining, cattle treated with insecticides need to be added to address these challenges. Malaria vaccine development is witnessing different challenges. RTS S/AS01 the most advanced candidate vaccine has shown low efficacy and faces some safety concerns especially for young children hence, limiting performance of this tool for eliminating malaria in intense transmission settings [255, 256]. So far malaria prevention through the use of vector control measures is considered to be highly cost-effective than other control interventions (e.g. mass drug administration) and scale-up has to be prioritized in all scenarios [257]. Indeed, the large scale-up of LLINs and IRS is considered to have averted an estimated 663 million clinical cases of malaria worldwide between 2001 and 2015 [258]. This highlights the central role that vector control has to play in the elimination of malaria. Thus, in order to achieve malaria elimination, the core interventions alone may not be sufficient. Adopting an integrated control approach is becoming critical for sustainable control of malaria in Cameroon. The combination of interventions suited for each epidemiological setting such as LLINs with larval source management or wall lining could be indicated for hyperendemic stratum experiencing high insecticide resistance. On the other side, combinations associating LLINs to spatial repellents could be indicated for hyperendemic stratum with outdoor malaria parasite transmission. In hypoendemic settings with limited breeding habitats or less diversity in vector species, the use of LLINs together with larval source management (LSM), attractive toxic sugar baits (ATSB) or gene drive could be indicated. In stratum displaying seasonal malaria parasite transmission, the use of LLINs with IRS spraying could be indicated for also managing insecticide resistance. New tools such as new generation nets or new generation IRS now available could be deployed to replace former LLIN formulations or to sustain control efforts. Although this has not been included in the present review, strengthening the health care system is part of the global effort for insuring sustainable malaria elimination. Because most of the current interventions are affected by operational challenges capacity building at different levels (local, community or national level) becomes central to ensure appropriate implementation of operational actions on the field. It is also relevant to stress the need for collaboration or networking to address capacity building issues. In this regard it could be indicated to revisit the achievement of the African Network on Vector Resistance to insecticides (ANVR) who acted between 2000 and 2004. During its short live period, the ANVR network was able to update and develop technical documents, standardize protocols for testing malaria vector susceptibility, provide guidelines for insecticide resistance management and contribute to capacity building. The revamping of such network and extending its actions to vector bionomics and vector control could be determinant to foster global elimination efforts across the sub-regions and sub-Saharan Africa. In the perspective of malaria elimination in Cameroon, it is important that challenges actually affecting control interventions be better identified and understood, and only intervention strategies tailored to be amenable to defined local epidemiological settings be taken into consideration. However, in each case, the deployment of any intervention should be accompanied by stringent routine entomological and epidemiological surveillance activities to monitor the success of the intervention and inform policy in real time.

## Data Availability

The datasets supporting the findings of this article are included within the article.

## Abbreviations

LLINs: long-lasting insecticidal nets
IRS: indoor residual spraying
WHO: World Health Organization
NMCP: national malaria control programme
HCH: hexachlorocyclohexane
ITN: insecticide treated nets
LSM: larval source management
ANVR: African Network on Vector Resistance to insecticides
OCEAC: Organization for the Coordination of the fight against Endemic diseases in Central Africa
